# Diffusion Tensor Imaging Group Analysis Using Tract Profiling and Directional Statistics

**DOI:** 10.3389/fnins.2021.625473

**Published:** 2021-03-22

**Authors:** Mehmet Özer Metin, Didem Gökçay

**Affiliations:** Department of Health Informatics, Middle East Technical University, Ankara, Turkey

**Keywords:** diffusion tensor imaging, directional statistic, group analysis, tract profile, major depression

## Abstract

Group analysis in diffusion tensor imaging is challenging. Comparisons of tensor morphology across groups have typically been performed on scalar measures of diffusivity, such as fractional anisotropy (FA), disregarding the complex three-dimensional morphologies of diffusion tensors. Scalar measures consider only the magnitude of the diffusion but not directions. In the present study, we have introduced a new approach based on directional statistics to use directional information of diffusion tensors in statistical group analysis based on Bingham distribution. We have investigated different directional statistical models to find the best fit. During the experiments, we confirmed that carrying out directional statistical analysis along the tract is much more effective than voxel- or skeleton-guided directional statistics. Hence, we propose a new method called tract profiling and directional statistics (TPDS) applicable to fiber bundles. As a case study, the method has been applied to identify connectivity differences of patients with major depressive disorder. The results obtained with the directional statistic-based analysis are consistent with those of NBS, but additionally, we found significant changes in the right hemisphere striatum, ACC, and prefrontal, parietal, temporal, and occipital connections as well as left hemispheric differences in the limbic areas such as the thalamus, amygdala, and hippocampus. The results are also evaluated with respect to fiber lengths. Comparison with the output of the network-based statistical toolbox indicated that the benefit of the proposed method becomes much more distinctive as the tract length increases. The likelihood of finding clusters of voxels that differ in long tracts is higher in TPDS, while that relationship is not clearly established in NBS.

## Introduction

Diffusion tensor imaging (DTI) can reveal complicated structural differences in patient groups by using the orientation and integrity of white matter tracts to identify white matter abnormalities. The diffusion tensor is the covariance matrix of diffusion coefficients calculated from gradient directions for each voxel. Although DTI is by nature a nonscalar image which provides directional information for the neural tracts, group-based DTI analyses are mainly conducted using scalar descriptors such as fractional anisotropy (FA) (Basser, 1995), relative anisotropy (RA) (Basser and Pierpaoli, 2011), axial diffusivity (AD), and radial diffusivity (RD) (Song et al., 2002). Such scalar metrics do not describe the full tensor shape or distribution and do not capture all of the information available in the data. By developing advanced metrics for connectivity analysis between groups of subjects in a nonscalar fashion, findings regarding abnormalities can be improved.

The principal diffusion direction (PDD), which is the eigenvector that corresponds to the largest eigenvalue of the tensor, captures the estimation of the fiber direction within the voxel. PDD has been used mainly in directionally encoded color (DEC) maps (Pajevic and Pierpaoli, 1999) which facilitate visual comparison but not quantitative group analysis. In order to evaluate PDD, which is a vector, statistical methods that analyze vector and tensor data are needed.

Directional statistics is conducted on vectors and directions based on observations on compact Riemannian manifolds (Pennec, 2006). Hence, it can encapsulate much more information than scalar metrics about the diffusion. Without the limitation of scalar statistics, one can evaluate dispersion and coherence values among the populations, fit directional model to the data, and perform hypothesis testing for group-based studies.

In the literature, directional statistics have been used to characterize fiber orientation distribution functions, to estimate fiber dispersion quantitatively *via* fanning and bending fiber geometries throughout the brain (Sotiropoulos et al., 2012; Tariq et al., 2016). In addition, directional statistics have also been utilized to extract bundle-specific metrics from crossing fiber models (Riffert et al., 2014) and fiber tractography (Parker et al., 2003). However, Watson distribution, which has been used in previous directional statistics in group analysis, contains limited parameters (Schwartzman et al., 2005; Hutchinson et al., 2012). Watson distribution is a bimodal probability distribution on a two-dimensional unit sphere S2 in R3 which is symmetrical around mean direction, where each direction and its negative have the same probability. In our previous study (Metin and Gökçay, 2014), it has been shown that Bingham distribution better fits into PDD distributions for white matter tracts and improves the depiction of variability among subjects in anisotropic tensors areas, such as fiber crossings. This is because Bingham distribution is a generalization of Watson distribution: it is bimodal and elliptic around mean direction.

Group analysis methods on DTI or DWI data can be classified into three: (1) region of interest (ROI)-based methods, (2) voxel-based analysis, and (3) fiber tract-based analysis. ROI-based methods are very labor intensive plus error-prone. On the other hand, voxel-wise comparison is open to misalignment of voxels because during registration of individual subject’s data to a common space, topological variabilities may not be thoroughly resolved (Jones and Cercignani, 2010) for each fine structure. The amount of smoothing can greatly affect the final results, but there is no principled way of deciding how much smoothing is “correct” (Jones et al., 2005). For instance, tract-based spatial statistics (TBSS) tackles the alignment and smoothing problem for voxel-wise statistics by combining strengths of VBM-style analyses and tractography-based approaches (Smith et al., 2006). In short, analyses that involve fiber tracts are contingent upon computation of quantitative parameters of interest along the tracts (Goodlett et al., 2009) within diffusion tensor images. The properties of the fiber tract can be scalar values derived from tensors such as MD, FA, or trace, as well as shape information such as curvature and torsion of the specific tract (Mandl et al., 2010).

In this study, we propose a new tract-based framework using directional information in diffusion tensors to improve statistical group analysis, named as track profiling and directional statistics (TPDS). For this purpose, we have (1) generated a new data structure called tract profile by clustering fibers across subjects and (2) developed a method based on directional statistics to compare white matter (WM) differences of different groups across each tract profile. Overall, this new DTI group analysis method is called TPDS.

In order to demonstrate the superiority of the proposed framework, we compared the tract profiling method with two widely used techniques: TBSS (Smith et al., 2006) and voxel-based analysis (VBA) (Hecke et al., 2009). Furthermore, we ran a third comparison with the network-based statistic (NBS) toolbox (Zalesky et al., 2010) which utilizes nonparametric statistical testing to identify the components of an *N* × *N* undirected connectivity matrix that differ significantly between two distinct populations.

As a proof of concept, we demonstrated the strength of TPDS in the identification of differences of structural connectivity in major depressive disorder in a small data set (*n* = 30). Although depression has traditionally been viewed as an affective disorder, the last few decades of research have shown that MDD is also associated with considerable disturbances in cognitive functioning, including executive functions, attention, memory, and psychomotor speed (Castaneda et al., 2008; McClintock et al., 2010). In MDD, multidimensional, systems-level differences are reported in discrete, but functionally integrated pathways (Mayberg, 2003). Therefore, differences in MDD can be expected to cover a wide range of WM tracts. So far, especially white matter disturbances and connectivity differences have been analyzed using DTI-based analysis in MDD (Seminowicz et al., 2004; Zou et al., 2008; Cullen et al., 2010; Kieseppä et al., 2010; McClintock et al., 2010; Helm et al., 2018). Most of these studies state that loss of integrity occurs in the WM fiber tracts of the frontal, temporal, and cingulate cortex of MDD patients. White matter integrity can be described as biophysical white matter changes as a result of microstructural characteristic in both intra- and extra-axonal environments of WM such as axonal water fraction (AWF), intra-axonal diffusivity, and extra-axonal axial and radial diffusivities. More specifically, reported abnormalities in the connectivity of the DLPFC and ACC circuits (Helm et al., 2018), as well as subcortical regions, complement other findings specified in affective disorders (Sexton et al., 2009).

## Materials and Methods

### Data Acquisition

In order to demonstrate the benefits of TPDS, we used T1-weighted, T2-weighted, and DTI MR data obtained from healthy subjects and patients with MDD.

#### Subjects

The control group consisted of 14 healthy subjects (8 female and 6 male) with age 31.71 ± 7.62, who had no history of neurological disease and also are not taking any medication. The depression group consisted of medication-naïve 16 subjects (8 female and 8 male, age: 31.12 ± 8.95)^1^. The data was collected as part of a local institutional project funded by METU (BAP-07-04-2012). Project management and subject recruitment were handled by a larger project^2^ for which the results will be published elsewhere.

#### MRI Parameters

Whole-brain MRI scans were collected using the Siemens MAGNETOM 3 T scanner situated at the Bilkent University UMRAM center. T1-weighted [repetition (TR): 2,500 ms, echo time (TE): 3 ms, inversion time (TI): 1,000 ms, flip angle (FA): 8°, sagittal plane 1 mm isotropic resolution], T2-weighted (TR: 5,900 ms, TE: 108 ms, FA: 120°, spacing: 2.2, slice thickness 2 mm), and DWII scans (TR: 8,270 ms, TE: 83 ms, FA: 90°, spacing: 2.2, seven images with *b*-factor = 0 s/mm^2^, 45 directions *b*-factor = 700 s/mm^2^) are collected from the participants in a single session.

### Data Processing

#### Pre Processing

**FIGURE 1:**
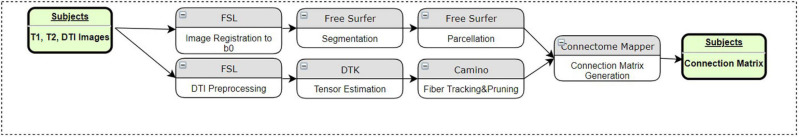
Components of the pre-processing pipeline before TPDS is performed.

We have implemented a fully automated pipeline to perform preprocessing as illustrated in Figure 1. The overall pipeline has been designed using the Connectome Mapper (Daducci et al., 2012). At the individual subject level, preprocessing steps are performed using several software toolkits. The first step is intrasubject registration of T1, T2, and DWI images using FSL’s FLIRT as described in Jenkinson and Smith (2001) and Jenkinson et al. (2012). The registration is first done between the T2-weighted image and DWI B0 images, and then the high-resolution T1-weighted image is registered to the T2-weighted image. To eliminate the problem of transforming diffusion tensors, all of the images are registered to the DWI B0 image. This way, all image operations are performed on the diffusion image. For segmentation and parcellation of ROIs, FreeSurfer (Fischl et al., 2002) has been used. These steps transform the subject’s MRI to uniform space and segment white and gray matter as well as cortical and subcortical structures based on the underlying atlas. The parcellation algorithm (Fischl et al., 2004) reveals 83 distinct cortical and subcortical structures of the brain using the Desikan–Killiany atlas (Desikan et al., 2006). All of these steps constitute the top row of Figure 1.

DTI processing begins with motion and eddy current artifact correction in FSL. Tensor estimation is done by Diffusion Toolkit (DTK) (Wang et al., 2007). For tractography (Parker et al., 2003; Cook et al., 2005), streamline fiber-tracking algorithm in Camino has been used. Each voxel in the parcellated image is selected as seeds. Eighty-three distinct cortical and subcortical areas are masked, and the generated binary image is used as the seed file of the algorithm for particular ROIs. For tracking, the fourth-order Runge–Kutta method has been chosen to propagate the tracks using a constant step size. Nearest-neighbor interpolation is applied around local voxel data. A minimum length criterion, 10 mm, is enforced to eliminate premature tract termination due to low SNR and low pathway anisotropy (Behrman-Lay et al., 2015). Each fiber bundle is pruned so that it only contains fibers connecting relevant regions. The number of streamlines depends on the size of the ROI. No additional elimination technique has been applied other than minimum length. These steps are illustrated in the second row of Figure 1.

Using the Connectome Mapper (Daducci et al., 2012), a connection matrix is generated to calculate the connectivity of the areas *via* the fiber tracts obtained in the first and second rows of Figure 1. After this step, the fiber tracts that connect corresponding brain areas will be bundled to construct relevant fiber bundles. In order to perform group analysis, one last step is necessary: the corresponding bundles of all subjects must be aligned. Therefore, both control and patient images are registered to the ICBM DTI-81 atlas using affine registration. The transformation obtained during this registration is applied to the fiber bundles as seen in the last row of Figure 1.

#### Tract Profiling

Tract profiles are cross sections of the fiber tracts that connect the ROIs specified by the connection matrix generated in preprocessing. For the connections in each ROI pair, a fiber bundle is formed based on the intersections of cross-sectional areas of all subjects’ DWI. Then, the medial line of the fiber bundle is computed. Finally, a cross-sectional profile is generated along the medial line so that the distribution of PDDs along each cross section is aggregated separately for each subject group.

##### Overlapping Fiber Calculation

**FIGURE 2:**
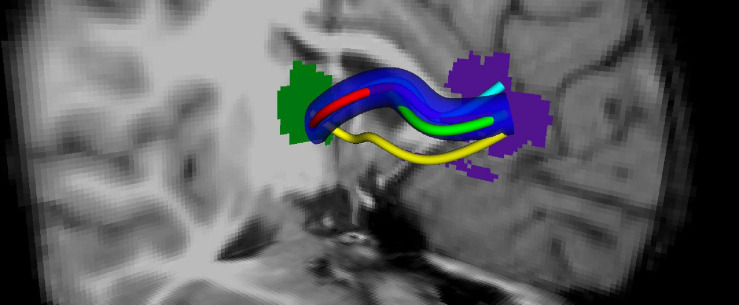
Tract profiling: Generation of an overlapping tract bundle between two ROIs (shown by green and blue) for all of the subjects regardless of the groups.

Overlapping fibers/voxels are calculated across all of the subjects. This is done for each fiber bundle by calculating its maximum overlap. During this process, some specific bundles might be left out as outliers. In Figure 2, the overlapping fiber bundle is shown between the two ROIs: thalamus (green) and rostral anterior cingulate (purple). The bundles shown with the yellow, cyan, green, and red colors are marked as outliers and left out of the overlapping area.

The voxel image can be represented as image 𝒫 where 𝒫 = (*Z*^3^, *m*, *n*, *B*) (Kong and Rosenfeld, 1989). Each element in *Z*^3^ is called a point of 𝒫 and each point in *B*⊆*Z*^3^ is called a black point and assigned 1. Each point in *Z*^3^\*B* is called a white point and assigned 0. *m* holds black points and *n* holds white points.

In order to be used in multisubject analysis, adaptation of this definition can be made as follows. For given ROI pairs (*i,j*), let 𝒫_0_, 𝒫_1_, …, 𝒫_*K*_ be a set where *K* is the number of subjects, and 𝒫_*k*_ is the fiber bundle image from subject *k*. A point in *P* is assigned as black point if and only if it is also black point for all sets in 𝒫_0_, *P*_1_, …, 𝒫_*K*_ for a given ROI(*i,j*).

##### Medial Line Generation

The skeleton of the overlapping bundles is calculated. The curve skeleton is a one-dimensional set which runs through the center of the overlapping bundles in such a way that it preserves the topological properties of the overlapping area. Connectivity conditions are defined as follows. The sequence of points (*x*_0_, *x*_1_, …, *x*_*n*_) is a *j*-path of length *n* ≥ 0 from the point *x*_0_ to point *x*_*n*_ in a nonempty set of points *X* if each point of the sequence is in *X* and *x*_*i*_ is *j*-adjacent to *x_*i*_*_–__1_ for each 1 ≤ *i* ≤ *n*. The adjacency can be defined as *N*_*j*_(*p*) the set of points *j*-adjacent, to the point *p*, where *j* = 6, 18, 26. Connectivity can be defined as *j*-connected if there is a *j*-path between them in *X*.

In order to construct the aforementioned skeleton, first of all, curve thinning (Blum, 1967; Kong and Rosenfeld, 1989) is used on *P*. The medial line of the fiber bundles was generated as depicted in Palágyi et al. (2001). As such, in each iteration, border points of *P* were deleted until no more deletion was possible. The algorithm is implemented as sequential iterations where each step checks for six subroutines for each of the six-directions that are immediate neighbors of a black point in *P*. In each iteration, border points are deleted upon satisfying a condition called simple point condition^2^. In this way, the object is shrunk uniformly in each direction. The operation is continued until no more shrinking is possible for each direction. By adding connectivity conditions, the skeleton ends up with the medial line in the near center of the object. In Figure 2, the example medial line for the fiber bundle is shown with dark blue.

Finally, the resulting medial line is smoothed by generating a *b*-spline representation as follows. In order to generate *b*-spline representation of the medial line, the voxel coordinates on the medial line are represented as data points {*P*_*k*_}, *k* ∈ MedialLine. A *b*-spline curve that fits the data is parameterized by *t* ∈ [0,1], where $X\,\left(t\right)=\sum_{i=0}^{n}U_{i,d}\,\left(t\right)\,Q_{i}$, the control points *Q*_*i*_ are unknown quantities that have been evaluated using the least-squares fitting method described below:

For *n* control points $\hat{Q}=\left[\begin{array}{c} Q_{0}\\ Q_{1}\\ \begin{array}{c} \vdots \\ Q_{n} \end{array} \end{array}\right]$, and *m* sample points $\hat{P}=\left[\begin{array}{c} P_{0}\\ P_{1}\\ \begin{array}{c} \vdots \\ P_{m} \end{array} \end{array}\right]$, the least-square error function between the *b*-spline curve and the sample points is the scalar valued function:

$$E(\hat{Q)}=\frac{1}{2}\sum_{k=0}^{m}|\sum_{j=0}^{n}U_{j,d}\left(t_{k}\right)Q_{j}-P{|}^{2}$$

To minimize the error function, *E*, where it is quadratic in the components of $\hat{Q}$, it is a graph of a paraboloid, so it has global minimum that can be found when all its first-order derivatives are 0. The first-order partial derivatives can be written as control points, *Q*_*i*_

$$\frac{\partial E}{\partial Q_{i}}=\sum_{k=0}^{m}\left(\sum_{j=0}^{n}U_{j,d}\,\left(t_{k}\right)\,Q_{j}-P_{k}\right)\,U_{j,d}\,\left(t_{k}\right)$$

$$\frac{\partial E}{\partial Q_{i}}=\sum_{k=0}^{m}\sum_{j=0}^{n}U_{i,d}\,\left(t_{k}\right)\,U_{j,d}\,\left(t_{k}\right)\,Q_{j}-\sum_{k=0}^{m}U_{i,d}\,\left(t_{k}\right)\,P_{k}$$

It can be written as $\sum_{k=0}^{m}\sum_{j=0}^{n}a_{k,i}\,a_{k,j}\,Q_{j}-\sum_{k=0}^{m}a_{k,i}\,P_{k}$, where *a*_*k*,*i*_=*U*_*k*,*d*_(*t*_*k*_) for 0≤ *i* ≤ *n*, by setting the partial derivatives to zero vector, and it leads to the system of equations:

$$0=\sum_{k=0}^{m}\sum_{j=0}^{n}a_{k,i}\,a_{k,j}\,Q_{j}-\sum_{k=0}^{m}a_{k,i}\,P_{k}=A^{T}\,A\,\hat{Q}-A^{T}\,\hat{P}$$

Where *A* = [*a*_*rc*_] is a matrix with *m* + 1 rows and *n* + 1 columns.

$$\hat{Q}={\left(A^{T}\,A\right)}^{-1}\,A^{T}\,\hat{P}=\left[{\left(A^{T}\,A\right)}^{-1}\,A^{T}\right]\,\hat{P}=X\,\hat{P}$$

Since *A* is tridiagonal where it has a contiguous set of upper bands and lower bands, the equation can be solved with the Cholesky decomposition and the vector of control points $\hat{Q}$ can be found.

Since derivative of spline is 1 less order of yet another *b*-spline where new control points are defined as $Q_{i}=\frac{p}{u_{i+1+1}-u_{i+1}}\,\left[c\,p\,s\,b\,r\,e\,a\,k\right]\,\left(P_{i+1}-P_{i}\right)$ from the surface tangent, a normal vector has been computed and cross-sectional areas have been extracted.

##### Calculation of Tract Cross Sections

**FIGURE 3:**
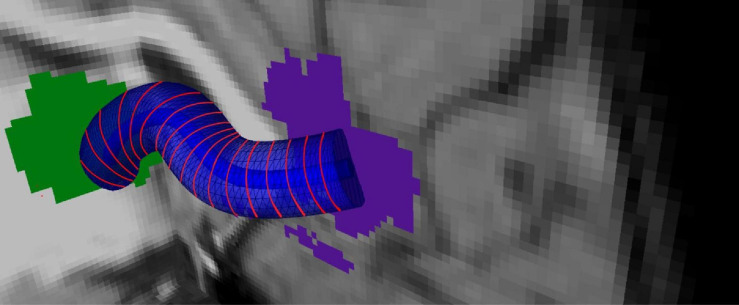
Tract profiling: Representation of the medial line of the overlapping bundle with b-splines and generation tract profiles.

**FIGURE 4:**
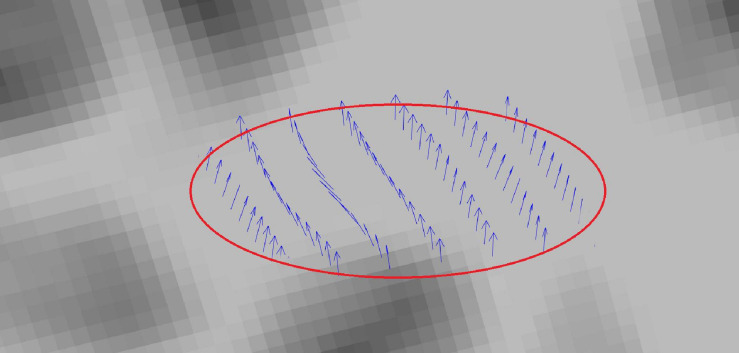
Tract profiling: Illustration of the PDDs from a single subject in a sample tract profile.

The skeleton is sliced with 2-mm regular intervals so that cross-sectional areas that are perpendicular to the *b*-spline are obtained using normal vectors computed from the surface tangents in Figure 3. For each voxel in *P* that intersects with these cross-sectional areas, PDDs that represent individual subjects are added as tract profiles representing that slice. Hence, for a tract with *J* slices, there are *J* tract profiles that contain PDDs which are representative of the subject group. An example tract profile (i.e., a slice with PDDs) from a single subject is shown in Figure 4. The PDDs from the subjects for a specific group are aggregated as follows. At each slice, there are fixed number of voxels, and at each voxel, there can be multiple PDDs, each coming from a different subject, depending on whether the subject’s tract goes through that voxel or not.

#### Directional Statistics

Statistical analysis is executed exclusively on areas that are defined by tract profiles eliminates voxel-wise comparison. Hence, misalignment problems no longer exist. Hypothesis testing is conducted only at cross-sectional tract profiles that are separated by 2-mm regular intervals. For the set of PDDs embodied in each tract profile *j*, a parametric directional statistic distribution is fitted. Through such parametrization, the PDDs of all subjects that fit into the tract profile *j* are projected onto a sphere.

**FIGURE 5:**
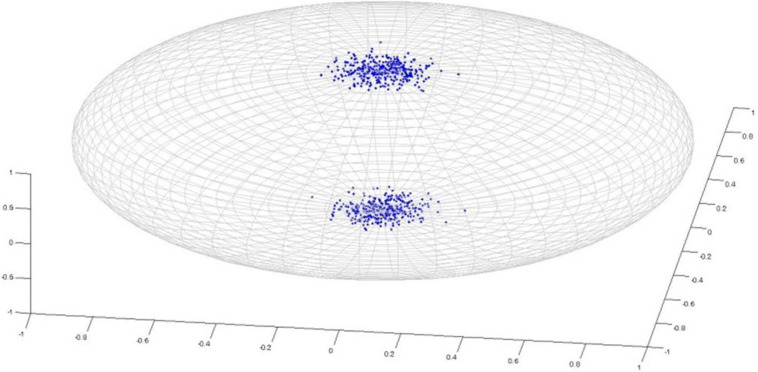
Watson Distribution.

**FIGURE 6:**
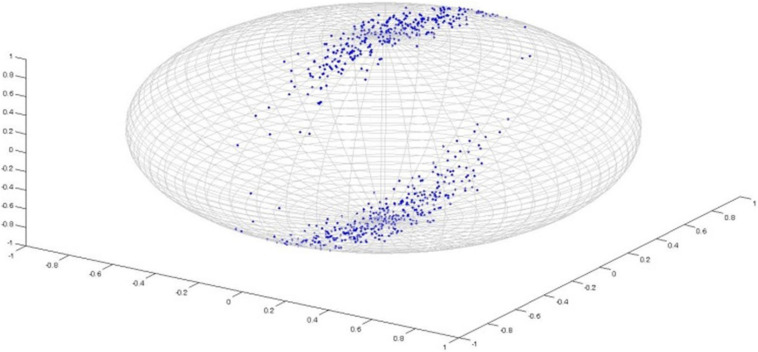
Bingham Distribution.

Watson distribution in Figure 5 is bimodal and symmetrical around mean direction. Watson distribution assumes that diametrically opposite points have the same probability. Also, the probability density function of axial distributions process antipodal symmetry [i.e., *f*(−*l,−m*,−*n*) = *g*(*l,m,n*)]. The probability distribution of random vectors that belong to the Watson’s family is spherical on a sphere. Directional statistics have been used in the analysis of DTI previously (Schwartzman et al., 2005; Hutchinson et al., 2012), and it has been shown that DTI principal direction analysis using directional statistics can better identify the differences in anatomic structure between populations compared with statistical tests of scalar values such as FA. Both of these studies used Watson distribution to analyze principal directions. On the other hand, Bingham distribution (Figure 6) is bimodal and elliptical (Fisher et al., 1993; Cheng et al., 2014). Bingham distribution is free from symmetrical constrains; hence, it provides more advanced distribution fitting options in comparison with Watson distribution.

*Watson distribution* is defined as follows (Mardia and Jupp, 1999):

    $\mathrm{Watson}\,\mathrm{Distribution}\,W_{p}\,\left(x;\mu ,\kappa \right)=c_{p}\,\left(\kappa \right)\,e^{\kappa \,{\left(\mu^{T}\,x\right)}^{2}};$

    $c_{p}\,\left(\kappa \right)=\frac{\text{Γ}\,\left(\frac{p}{2}\right)}{2\,\pi^{p/2}\,M\,\left(\frac{1}{2},\frac{p}{2},\kappa \right)}$

where *x* is the unit random vector, μ is the mean vector, is the concentration value, *M* is Kummer’s confluent hypergeometric function, Γ is a gamma function, and *p* is the dimension of the distribution. To estimate maximum likelihood of this function, we take logarithm. Hence, the log-likelihood function is

$$l\,\left(\mu ,\kappa \pm x_{1},\ldots ,\pm x_{n}\right)=\kappa \,\sum_{i=1}^{n}{\left(x_{i}^{T}\,\mu \right)}^{2}-n\,\text{log}\,M\,\left(\frac{1}{2},\frac{p}{2},\kappa \right)$$

$$=n\,\left\{\kappa \,\mu^{T}\,\bar{T}\,\mu -\text{log}\,M\,\left(\frac{1}{2},\frac{p}{2},\kappa \right)\right\}$$

where $\bar{T}$ is the scatter matrix of the given data. Differentiation with respect to κ gives

$$D_{p}\left(\kappa \right)={\hat{\mu }}^{T}\bar{T}\hat{\mu };\text{for}p=3;=\frac{M\,\left(1.5,3.5,\kappa \right)}{3^{*}\,M\,\left(0.5,1.5,\kappa \right)}.$$

And to find its maximum likelihood estimate, we need a derivative of *D*_*p*_(κ) for *p* = 3

$$D_{3}^{\prime }=\frac{M\,\left(2.5,3.5,\kappa \right)}{5\,M\,\left(0.5,1.5,\kappa \right)}-\frac{1}{9}*{\left(\frac{M\,\left(1.5,2.5,\kappa \right)}{M\,\left(0.5,1.5,\kappa \right)}\right)}^{2}.$$

The Newton–Raphson method can be used tfind maximum values for *D*_*p*_(κ) and the biggest eigenvalue of scatter matrix, *t*_1_, for a bipolar distribution or *t*_3_ for a girdle distribution.

*Bingham distribution* is defined as a trivariatnormal distribution on a unit sphere. Different from Watson distribution, it has three orthogonal directions as μ_1_, μ_2_, μ_3_ and concentration values (κ_*n*_)for each orientation vector (Watson and Williams, 1956).

Concentration values define the dispersion of the distribution, where

(1) κ_1_=κ_2_ = 0 results in a spherical distribution of axes.
(2) κ_1_=κ_2_≪0 results in a symmetric bipolar distribution.
(3) κ_1_ < κ_2_≪0 results in an asymmetric bipolar distribution.
(4) κ_1_≪κ_2_ < 0 results in an asymmetric girdle distribution.
(5) If κ_1_≪0*and*κ_2_ = 0, then Watson distribution is obtained.

The probability distribution function of Bingham distribution is defined as follows (Bingham, 1974):

    $\mathrm{Bingham}\,\mathrm{Distribution}\,B_{p}\,\left(x;K\right)=c_{p}\,\left(K\right)\,e^{x^{T}\,K\,x};$

    $c_{p}\,\left(K\right)=\frac{\text{Γ}\,\left(\frac{p}{2}\right)}{2\,\pi^{p/2}\,F\,\left(\frac{1}{2},\frac{p}{2},K\right)}$

where *x* is the unit random vector, *K* is the 3 × 3 orthogonal orientation matrix with concentration values, *F* denotes the confluent hypergeometric function of matrix argument, Γ is the gamma function, and *p* is the dimension of the distribution. For a given random sample ±*x*_1_,…,±*x*_*n*_, the log-likelihood function can be written as:

$$l\,\left(K;\pm x_{1},\ldots ,\pm x_{n}\right)=n\,\left\{\log t\,r\,\left(A\,\bar{T}\right)-\text{log}\,F\,\left(\frac{1}{2},\frac{p}{2},K\right)\right\}$$

We can write *K* and $\bar{T}$ in polar form as = *UKU*^*T*^, $\bar{T}=V\,t\,V^{T}$ with *U* and *V* being orthogonal. *K* = diag(κ_1_,…,κ_*p*_) and $t=\left({\bar{t}}_{1},\ldots ,{\bar{t}}_{p}\right)$, where κ_1_≥…≥κ_*p*_ and ${\bar{t}}_{1}\geq \ldots \geq {\bar{t}}_{p}$. As suggested by Bingham himself, the following approximations can be used.

For the bipolar case:

    $d={\bar{t}}_{2}-{\bar{t}}_{3},s={\bar{t}}_{1}+{\bar{t}}_{2},\kappa_{0}=-D_{3}^{-1}\,\left({\bar{t}}_{1}\right)$

    $\kappa_{1}\approx 0,\kappa_{2}\approx \kappa_{0}+\delta ,\kappa_{3}=\kappa_{0}-\delta$

For the girdle case:

    $d={\bar{t}}_{1}-{\bar{t}}_{2},s={\bar{t}}_{1}+{\bar{t}}_{2},\kappa_{0}=-D_{3}^{-1}\,\left({\bar{t}}_{3}\right)$

    $\kappa_{1}\approx 0,\kappa_{2}=-2\,\delta ,\kappa_{3}=\kappa_{0}-\delta$

where $\delta =\frac{2\,d\,\kappa_{0}}{s\,\left(\kappa_{0}-1.5\right)+1}$

**FIGURE 7:**
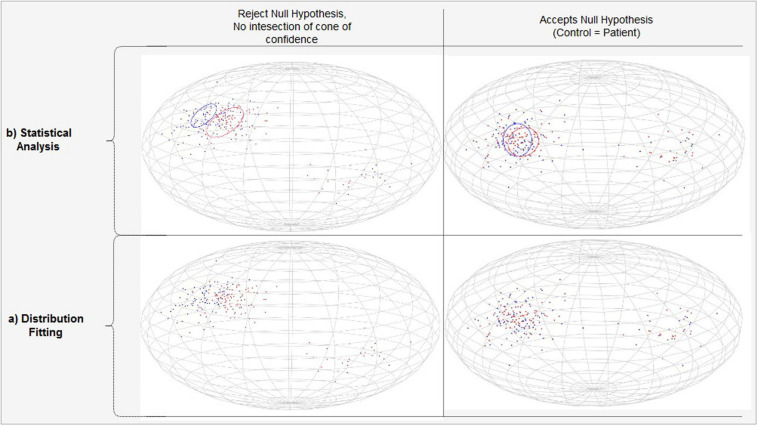
PDD projections modeled by the Bingham Distribution. **(A)** Separated (Left) versus overlapping (Right) vector projections of PDDs on unit sphere for two different subject groups shown with blue and red. **(B)** Statistically significant (Left) versus insignificant (Right) differencs between populations.

After parametric representation through either Watson or Bingham distribution, two group of subjects can be compared by using an eclipse of confidence defined by the *p* value. For fitting a single group’s data, the mean direction vector of the group is computed. If it lies inside the eclipse of confidence of the targeted distribution, then the null hypothesis is likely, justifying a reasonable fit to the associated directional distribution. On the other hand, if the confidence ellipse around the mean direction does not overlap for a given confidence level, then the null hypothesis is unlikely, rejecting the fit. For two groups, the case with different means is indicated by separated cones of confidence, which in turn indicates significant differences. On the other hand, overlapping cones of confidence indicate insignificant differences, hence acceptance of the null hypothesis. An example distribution is provided in Figure 7, for two groups of subjects, for representation with the Bingham distribution.

Details of the eclipse of confidence can be given as follows. The maximum likelihood estimates of concentration parameters κ_1_,κ_2_ can be obtained from maximizing the log-likelihood function, where *w*_*n*_ are the eigenvalues of the principal eigenvector of the orientation matrix:

$$F=-N\,\text{log}\,\left(4\,\pi \right)-N\,\log\,d\,\left(\,\kappa_{1},\kappa_{2}\right)+\kappa_{1}\,w_{1}\,\kappa_{2}\,w_{2}$$

Maximum likelihood estimators of κ_1_,κ_2_ in the Bingham distribution for given eigenvalues *w*_1_,*w*_2_ can be estimated as calculated by Mardia and Zemroch (1977).

The confidence ellipse around the mean direction within the specified percentage (%) of the estimated concentration values of distribution as

$$e_{\%}^{m\,n}=\sqrt{\left[\frac{X_{\%}^{2}}{2\,N\,\left(\Delta_{m\,n}\right)}\right]}\,\text{for}\,\Delta_{m\,n}=\left(\kappa_{m}-\kappa_{n}\right)\,\left(w_{m}-w_{n}\right)\,\text{and}\,X_{\%}^{2}$$

is the chi-squared value for two degrees of freedom and % is *p* value for confidence interval.

For *p* = 0.01 and having κ_*3*_ = 0 (Fisher et al., 1993) ends up with the semi-axes of the confidence eclipse about the mean direction associated with *w*_*3*_ as below:

$$e_{32}=-1.517\,\frac{1}{k_{2}N(w_{3}-w_{2)}}\,\text{and}\,e_{31}=-1.517\,\frac{1}{k_{1}N(w_{3}-w_{1)}}$$

### Performance Analysis

We have conducted two performance tests to analyze the effectiveness of the proposed method. First, we have analyzed the models generated by TPDS in comparison with VBA and TBSS. Hereby, we have adapted directional statistics to TPDS, TBSS, and VBA to compare their overall efficiency in representing vector-based statistical models. This test aimed to show the efficiency of tract profiling over voxel-based and skeleton-based analysis. Second, we have applied the full TPDS algorithm to the two subject populations (i.e., MDD versus healthy controls) and compared the results with NBS. This test aimed to show the efficiency of combining tract profiles with directional statistics over conventional methods. In this test, the effects of fiber length in estimating group differences were also evaluated.

#### Analysis of the Strengths of VBA, TBSS, and TPDS in Tract Modeling

**FIGURE 8:**
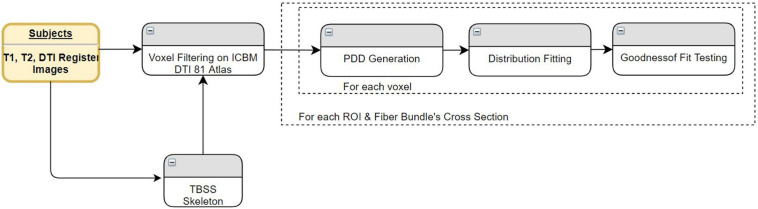
Comparison of VBA/TBSS with TPDS data processing pipeline.

In this test, we used a single group (i.e., healthy subjects). The statistics were derived using three different methods, VBA, TBSS, and TPDS, only on white matter areas—not using GM ROIs. As seen in Figure 8, the white matter areas that have been segmented using FreeSurfer are mapped to ICBM DTI-81 atlas (Mori et al., 2008) to allow for intersubject data aggregation. For VBA analysis, the atlas-based white matter areas are overlayed for all subjects for further processing. For TPDS analysis, tract profiles are generated from the atlas mappings of all subjects. In TBSS, before performing atlas mapping, skelotonized areas are generated from individual subject tracts. The rest of the data processing pipeline is the same for all three methods. At the first step, for each WM ROI, based on which method is used for defining the tract, PDDs are generated. Then these PDDs are parametrically modeled by two separate directional distributions, namely Bingham and Watson. Finally, in the last step, several PDDs are generated to represent the entire group using the newly developed parametrical models, and goodness of fit is computed to evaluate how good the chosen model is.

##### PDD Generation

For each subject, primary diffusion directions are extracted for each voxel inside the given WM area using the primary eigenvector of the diffusion tensor. The WM area differs based on the chosen representation. In VBA, the WM area is extracted based on segmentation of the specific WM ROI. In TBSS, it is based on the skeleton of the tract in the WM ROI. In TPDS, it is embodied within each tract profile that composes the entire tract in the WM ROI. Aggregated data from all subjects compose the data to be fitted for each WM area.

##### Distribution Fitting

Watson and Bingham distributions were fitted to model each tract using the maximum likelihood method. For each tract, the parameters of the theoretical model were estimated from the pdf at hand. Then this theoretical probability density function was evaluated iteratively using synthetic random vector data for a total of 700 vectors that were almost uniformly distributed along a sphere. Finally, the difference between the estimated pdf and the random pdf is tested for null hypothesis.

##### Goodness of Fit Testing

Pearson’s chi-square tests have been used for goodness of fit tests to evaluate whether the observed frequency distribution differs from the theoretical distribution. Comparison of distributions is done using ANOVA and the chi-square test statistics was also used for each ROI.

In order to apply Pearson’s chi-square tests to check whether the observed frequency distribution differs from a theoretical distribution, the following steps are applied on the original data and synthetic random vector data and the respective models.

(1) For Watson distribution, the sample mean direction, $\bar{R}$, has been evaluated as a regular vector sum of the vectors under a population of vectors. The mean direction is a unit vector that is in the same direction with *R*: $\bar{x}=\frac{\sum_{i}x_{i}}{R}$, $\bar{y}=\frac{\sum_{i}y_{i}}{R}$, $\bar{z}=\frac{\sum_{i}z_{i}}{}\,R$.
(2) For Bingham distribution, the axis of moment of inertia of sample, $\bar{t}$, has been evaluated using the scatter matrix of distribution *S*. For the bipolar case, it is the biggest eigenvector, and for girdle case, it is the smallest eigenvector.
(3) The transformations $\bar{\theta },\,\bar{\phi }$ have been evaluated in order to shift either $\bar{R}$ or $\bar{t}$. to positive *z*-axis.
(4) The transformation has been applied to original and synthetic data.
(5) The angle θ has been calculated as the angle between the positive *x*-axis and the projected vector on the *x*–*y* plane: 0 < θ < 2π.
(6) The observed frequencies and the excted frequencies were θ_1_ < θ < θ_2_, where the number frequency bins is 50 and θ_*2*_ – θ_1_ ≈7.2^o^.

#### Analysis of the Group Difference Maps Generated by NBS and TPDS

In this part, the proposed framework will be applied to test for differences of fiber tract profiles between MDD patients and control subjects. Based on the same fiber tracts and connectivity matrix for healthy volunteers, comparisons will be made with the results of the network-based statistics. For this purpose, we used the 83 × 83 connectivity matrix generated at the end of data preprocessing by the Connectome Mapper (Figure 1).

In NBS, for each group, each pairwise association (*i*,*j*) between ROI *i* and ROI *j* is treated separately. First, Fisher’s *r*-to-*z* transform has been applied to ensure normality. Then, the test statistic of interest—which is the normalized number of fiber bundles—is compared between the groups using *t*-statistic. In order to correct for multiple comparisons, permutation testing was used to select the *p* value controlled for the FWE for each connected component. For each permutation, the same threshold is applied to define a set of suprathreshold links of connected components. Suprathreshold and the number of permutations were set according to the default parameter settings of NBS with corrected *p* < 0.005.

In TPDS, the following procedure is repeated for each possible connection between distinct ROI pairs (i.e., 83 × 83 times divided by 2). Tract profiles between each ROI *i* and ROI *j* are extracted for the healthy and MDD groups. Then for each slice in the tract profiles, significance is tested with a threshold value of *p* < 0.005. If there are *n* contiguous slices that satisfy this, it is indicated that the connection between ROIs *i* and *j* is significantly different between the control and patient groups. It is possible that there are multiple clusters of *n* contiguous slices that satisfy this condition. In order to reflect this information, we prepared a new 83 × 83 connectivity matrix, which contained the number of significantly different clusters between the two groups that are compared. Therefore, the difference map that is achieved through TPDS reflects a weighted graph, weight being the number of significantly different clusters between the two groups for that particular *i* to *j* connection. The more the number of significantly different *n* contiguous slices, the more the weight of the difference map.

Selection of *n* must be done according to a criterion related to the plausible tract lengths. In order to eliminate premature tract termination that result from low SNR and low pathway anisotropy (Behrman-Lay et al., 2015), 10 mm is the shortest tract length to be considered. Since DTI image has 2.2 mm spacing, choosing *n* as 4 satisfies this constraint. In other words, at least four consecutive cross-sectional areas must be found within a fiber bundle where the PDD of each cross-sectional area belongs to significantly different Bingham distributions for the control and MDD groups.

## Results

The results of the performance tests that we performed to investigate the effectiveness of TPDS are as follows.

### Comparison of VBA and TBSS With TPDS Using Directional Statistics

As can be seen in Table 1, among VBA, TBSS, and TPDS, the best fitted distribution is more representative in TPDS because the goodness of fit scores are better according to *p* values. In addition, based on the results of TPDS, the Bingham distribution is reported to be more favorable than the Watson distribution because only 2 out of 48 white matter tracts are represented better with Watson. Obviously, it is evident that TPDS is a better alternative to represent tracts in comparison with VBA and TBSS, because it favors a more parametrical fit to the entire set of fiber tracts.

**TABLE 1:** Comparison of voxel-based analysis (VBA) and tract-based spatial statistics (TBSS) with tract profiling and directional statistics (TPDS) (VBA and TBSS have been adapted to run directional statistics).

WM tract	VBA model (*p* value)	TBSS model (*p* value)	TPDS model (*p* value)
Middle cerebellar peduncle	No fit (0.803)	No fit (0.425)	Bingham (0.021)
Pontine crossing tract	No fit (0.092)	Bingham (0.043)	Bingham (0.004)
Genu of corpus callosum	Bingham (0.030)	Bingham (0.032)	Bingham (0.007)
Body of corpus callosum	Bingham (0.001)	Bingham (0.001)	Bingham (0.031)
Splenium of corpus callosum	Bingham (0.046)	Bingham (0.036)	Bingham (0.045)
Fornix (column and body of fornix)	No fit (0.707)	No fit (0.135)	Bingham (0.017)
Corticospinal tract R	No fit (0.067)	No fit (0.087)	Bingham (0.048)
Corticospinal tract L	No fit (0.541)	Bingham (0.041)	Bingham (0.025)
Medial lemniscus R	No fit (0.706)	Watson (0.046)	Bingham (0.036)
Medial lemniscus L	No fit (0.278)	No fit (0.078)	No fit (0.090)
Inferior cerebellar peduncle R	Watson (0.019)	Watson (0.037)	Bingham (0.016)
Inferior cerebellar peduncle L	No fit (0.970)	No fit (0.570)	Bingham (0.019)
Superior cerebellar peduncle R	Watson (0.032)	Bingham (0.042)	Bingham (0.045)
Superior cerebellar peduncle L	Watson (0.026)	Bingham (0.044)	Bingham (0.012)
Cerebral peduncle R	No fit (0.064)	Bingham (0.044)	Bingham (0.023)
Cerebral peduncle L	No fit (0.078)	Bingham (0.032)	Bingham (0.022)
Anterior limb of internal capsule R	Watson (0.030)	No fit (0.079)	Watson (0.023)
Anterior limb of internal capsule L	Bingham (0.002)	Bingham (0.038)	Watson (0.025)
Posterior limb of internal capsule R	Watson (0.014)	No fit (0.067)	Bingham (0.008)
Posterior limb of internal capsule L	Bingham (0.017)	Bingham (0.033)	Bingham (0.033)
Retrolenticular part of internal capsule R	Watson (0.034)	No fit (0.074)	No fit (0.083)
Retrolenticular part of internal capsule L	Watson (0.015)	No fit (0.065)	No fit (0.106)
Anterior corona radiata R	No fit (0.278)	Bingham (0.012)	Bingham (0.045)
Anterior corona radiata L	Bingham (0.0012)	Bingham (0.002)	Bingham (0.001)
Superior corona radiata R	Watson (0.043)	Bingham (0.009)	Bingham (0.001)
Superior corona radiata L	No fit (0.165)	Bingham (0.035)	Bingham (0.019)
Posterior corona radiata R	Watson (0.002)	Bingham (0.017)	Bingham (0.002)
Posterior corona radiata L	Watson (0.001)	Bingham (0.019)	Bingham (0.006)
Posterior thalamic radiation R	Bingham (0.003)	Bingham (0.002)	Bingham (0.024)
Posterior thalamic radiation L	Bingham (0.006)	Bingham (0.002)	Bingham (0.009)
Sagittal stratum R	No fit (0.188)	No fit (0.488)	Bingham (0.032)
Sagittal stratum L	No fit (0.065)	No fit (0.265)	Bingham (0.047)
External capsule R	Bingham (0.006)	Bingham (0.006)	Bingham (0.001)
External capsule L	Bingham (0.001)	Bingham (0.001)	Bingham (0.001)
Cingulum (cingulate gyrus) R	Bingham (0.015)	Bingham (0.033)	Bingham (0.003)
Cingulum (cingulate gyrus) L	Bingham (0.002)	Bingham (0.001)	Bingham (0.001)
Cingulum (hippocampus) R	Watson (0.004)	Bingham (0.004)	Bingham (0.001)
Cingulum (hippocampus) L	No fit (0.118)	Bingham (0.019)	Bingham (0.041)
Fornix (cres)/stria terminalis	Bingham (0.04)	No fit (0.050)	Bingham (0.009)
Fornix (cres)/stria terminalis	Bingham (0.012)	No fit (0.128)	Bingham (0.005)
Superior longitudinal fasciculus R	Watson (0.002)	Bingham (0.043)	Bingham (0.021)
Superior longitudinal fasciculus L	Watson (0.025)	Bingham (0.040)	Bingham (0.011)
Inferior fronto-occipital fasciculus R	Bingham (0.025)	Bingham (0.008)	Bingham (0.003)
Inferior fronto-occipital fasciculus L	Bingham (0.004)	Bingham (0.062)	Bingham (0.002)
Superior fronto-occipital fasciculus R	Bingham (0.003)	Bingham (0.018)	Bingham (0.001)
Superior fronto-occipital fasciculus L	Bingham (0.044)	Bingham (0.026)	Bingham (0.006)
Uncinate fasciculus R	No fit (0.483)	No fit (0.091)	Bingham (0.003)
Uncinate fasciculus L	No fit (0.896)	Bingham (0.039)	Bingham (0.002)
Tapetum R	No fit (0.595)	No fit (0.092)	Bingham (0.092)
Tapetum L	No fit (0.535)	No fit (0.103)	Bingham (0.004)

A close inspection of Table 1 reveals that in terms of representing a given WM tract parametrically, TBSS is superior to VBA, and TPDS is superior to TBSS. It is evident that VBA contains more noise than TBSS and TPDS, because it contains the entire WM area from all subjects. Due to high noise, VBA fails to represent some of the tracts parametrically. On the other hand, TBSS is better than VBA, because it removes the areas—hence the noise associated in these parts—that lie outside the fiber bundles which constitute the skeleton. However, TBSS is not better than TPDS, because it smooths out the tracts while forming the skeleton and loses specificity. Overall, the tract profiles computed in TPDS are selective in choosing representative samples of the DWIs that are more informative, because outliers are removed while computing the medial line. Since the data points all belong to the same tract and on the same cross section over the medial line, very similar diffusion properties are expected for each analysis point. This tends to eliminate all negative effects of misalignment of images and partial volume effect. Due to this property, the computational effectiveness of TPDS is higher than other methods, because the model can be decided with much less number of data points.

The advantage of the Bingham distribution might be explained through the ease of fitting a girdle distribution in comparison with fitting a homogeneous mean direction distribution. The girdle distribution allows for more parameters; hence, it makes the development of a more general model possible. Furthermore, the computational accuracy of the Bingham distribution is better because the tracts represented with this distribution fit to the PDD of the actual tracts with a smaller *p* value.

### Comparison of the Group Differences in Connectivity Maps Using Network-Based Statistics and TPDS

**FIGURE 9:**
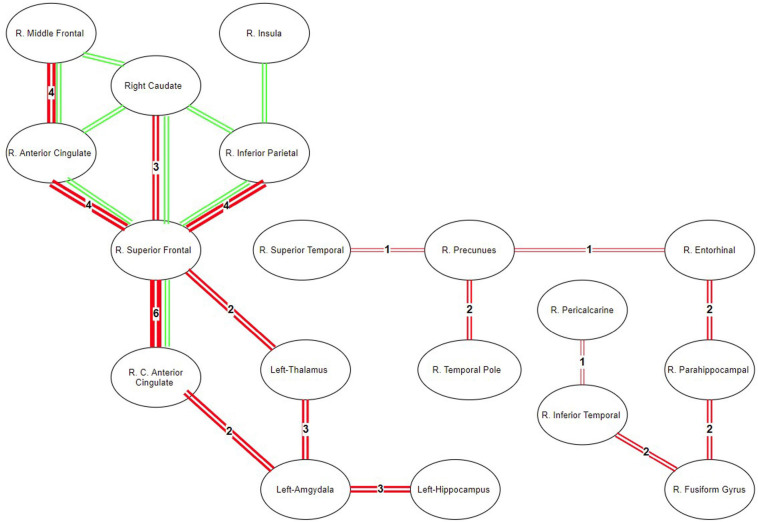
Map of ROIS with statistically different connectivity between control and patient groups. Green lines represent the common connections that are found different between the groups using NBS. Red lines represent the significantly different connections detected by the directional statistics using tract profiling.

In NBS, with corrected *p* < 0.005, seven regions and eight connections have been observed to contain lower FA in MDD. Particularly, the connections in the right hemisphere and between the superior frontal cortex and rostral/caudal components of the anterior cingulate cortex, caudate, and inferior parietal cortices had lower FA in MDD. These connections are shown in Figure 9 as green lines.

In TPDS, significantly different connections between the healthy and MDD groups are seen in Figure 9 as red lines. The thickness of the lines reflects the weights or in other words the number of cross-sectional areas above the threshold n (e.g., A weight value of 1 indicates that there exists only one slice cluster with significantly different *n* contiguous tract profiles, whereas a weight value of 6 indicates that there exist 6 disjoint clusters of *n* contiguous tract profiles that are significantly different). The right hemisphere differences reported by NBS, namely the frontal (superior frontal and rostral middle frontal) and medial (caudal and rostral anterior cingulate), are also detected by our method. But, additionally, TPDS revealed differences between the healthy and MDD populations in limbic, temporal cortex, occipital cortex, and hippocampal connections, as well as a few left hemisphere areas such as the amygdala, hippocampus, and thalamus.

The strength of the tract profile structure lies in the reduction of the misalignment problem. Furthermore, observations of the directional changes become more specific because contributions of the local changes can be reported along the tract not by the contribution of isolated voxels but by several slices across the two ROIs. Therefore, the proposed directional statistics comparison is expected to be a superior differentiator for especially long tracts.

**FIGURE 10:**
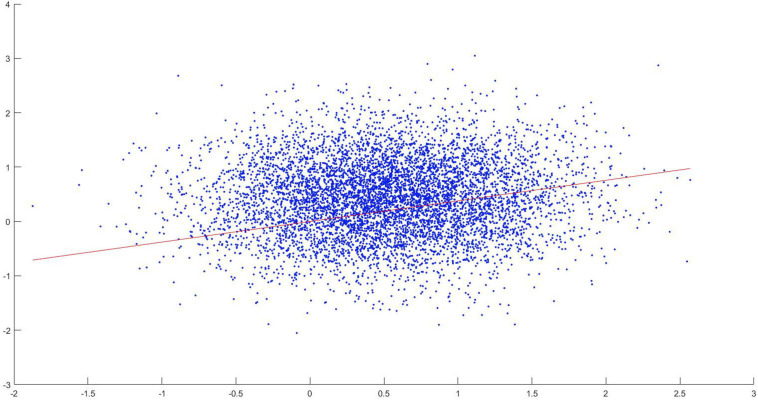
Scatter diagram of z-score of tract lengths versus significantly different clusters.

In order to verify this, the following analysis has been done. Using TPDS, for each tract connecting 83 different regions, the *z*-score of each length is plotted against the *z*-score of the number of significantly different profile slices. For this purpose, the maximum overlapping shape (skeleton) is used. When regression lines are fitted to investigate the relationship with tract length and the number of different clusters, it is seen that the likelihood of finding clusters of voxels that differ in long tracts increase with respect to path length. This has been also tested using a linear regression model, where it has been found that the *z*-score of tract length significantly correlated with the *z*-score of the number of significantly different profile slices (*p* < 0.05, adjusted *R*^2^: 0.00162) as seen in Figure 10. Although the effect size is small, we can indicate that TPDS is a powerful method to find differences in two populations, especially as the tract lengths get longer.

## Discussion

In this study, we proposed a novel framework for WM fiber connectivity analysis using TPDS. In contrast with other group studies (Goodlett et al., 2009) that are based on FA values, directional statistics deals with compact Riemannian manifolds, which allow observations regarding local diversities of principal diffusion directions of voxels in different groups of subjects.

### Comparison of TPDS With Other Techniques Used in Analysis of Groups of DWI

Our pipeline implementation can be regarded as quantitative tractography. We analyze diffusion properties on the exact tracts and derive the statistics over sample points taking neighborhood cells into consideration. A similar method has been offered by Corouge et al. (2006) where diffusion properties along the fiber tracts, called fiber property profiles, are extracted. In that study, fiber tract parameterization was based on arc length parameter, starting from each fiber’s intersection with an “origin” plane. Goodlett et al. also proposed a similar tract profiling approach, where diffusion properties are calculated along the tract for each fiber bundle (Goodlett et al., 2009). Our method introduces three main improvements to these quantitative tractography methods. First, we are not just limiting the method with known anatomical fiber bundles but can derive statistics from any pair of connected gray matter areas. Second, we have introduced skeletonization and pruning to allow for applying statistics only within common areas across the groups. Third, we introduced vector analysis using directional statistics over scalar analyses such as FA, MD, etc.

There exist other methods which use directional statistics in DTI (Schwartzman et al., 2005; Hutchinson et al., 2012). However, these methods analyze group differences based on ROIs, not fiber tracts, ignoring the underlying connectivity. We have devised the tract profiling algorithm to operate on relevant voxels among the fibers that connect each ROI obtained from fully automatic brain segmentation and parcellation. Local registration errors are reduced after calculating cross-sectional area of the fibers and finding medial lines (i.e., profiles) to continue tract analysis. Afterwards, Bingham distribution, which is the most general form of directional distribution, is used for tract-based directional analysis, ensuring minimum parametric assumptions about the dataset. To the best of our knowledge, this approach has not been implemented in group analysis of DTI before.

Neurite orientation dispersion and density imaging (NODDI) is a novel neurite imaging and analysis framework and provides sensible neurite density and orientation dispersion estimates. Unlike FA, NODDI analyzes density and orientation dispersion separately. NODDI uses orientation distribution function (ODF), defined as Watson distribution which constrains the dispersion about the dominant orientation (Zhang et al., 2012). However, Bingham distribution fits better to diffusion properties, in comparison with Watson. Bingham-NODDI extends the NODDI method by generalizing it with Bingham distribution to cover anisotropic orientation dispersions of neurites (Tariq et al., 2016). Similarly, in our study, we found that modeling ODF using Bingham distribution explains the data better regardless of the tract identification, be it through VBA, TBSS, or our method, TPDS. A major difference between our approach and NODDI is in the estimation of the dispersion. The modeling we used to implement the Bingham distribution estimates dispersion in the vicinity of the dominant orientation, separately for the primary and secondary dispersion orientations. This eliminates the key limitation of NODDI, failing to model complex neurite configurations such as those arising from fanning and bending axons. On another front, just like ours, orientation dispersion (ODI) generated by the NODDI method can be also used with the TBSS method instead of FA metric (Timmers et al., 2016; Taoka et al., 2020). In this aspect, the main difference between our method and NODDI is in extending fiber dispersion along the tracts that connect two ROIs. By allowing such extension, our method enables using fiber dispersions as track characteristics and analyzing disease-related effects on connectivity of the tracks rather than the voxel.

We have demonstrated that in addition to scalar diffusibility changes, analyzing principal diffusion directions along a tract detects local changes better than scalar values. The strength of the directional statistics-based analysis we proposed lies in its applicability to TBSS and VBA as well; it is not limited to tract profiles.

Voxel-based analysis needs to register the subject’s images to a common coordinate frame. However, the fiber tracts do not accurately align during this process due to variation in tract size and shape. Especially, long-range fiber tracts contain more shape variation across subjects (Wassermann et al., 2011), so they are more prone to such misalignment. This problem is still valid for TBSS because even the voxel skeletons do not ensure that all relevant voxels correspond to the same tract (de Groot et al., 2013).

In directional statistics, the misalignment problem though the tract becomes more critical compared with scalar statistics like FA. As seen from the results of the first set of performance tests, tract profiles are superior structures for resolving the shape differences in comparison with VBA and TBSS, because tract profiles are better in terms of fitting a model to PDD vectors. We investigated the goodness of fit characteristics of VBA and TBSS, respectively, on all WM areas and on skeletonized WM areas using directional statistics. We found that several tracts in VBA and TBSS are rejected to fit to the most general Bingham distribution which contains minimum assumptions about the data. In comparison when tract profiling is used, most tracts could be fit parametrically, except a few. A parametrical model is advantageous in data processing, since it facilitates population-based comparisons.

The aforementioned tests also show how directional statistics can be adapted to the widely used analysis methods such as TBSS or VBA. Instead of FA values, PDD vectors can be used over each voxel within the skeleton. FA metric uses eigenvalues of the underlying diffusion characteristics of the voxel and defines only the amount of diffusion asymmetry where PDD uses the first eigenvector of the diffusion characteristic. The FA metric is sensitive to the underlying fiber architecture and correlates with PDD changes in disease conditions. However, FA does not have direction property. Different orientations might result in the same FA value simply because orientational changes of the diffusion property of the voxel might not end with FA changes, when there is a difference in eigenvector orientation but not its value. So, the FA metric is not as sensitive as PDD in detecting diffusion characteristic differences along the fiber track. As can be seen in Table 1, Bingham distribution fits better to describe the differences in the majority of white matter tracks. Further studies should be conducted to ease adaptation of directional statistics to TBSS skeletons and also to resolve issues related to the multiple comparison problem.

PDD analysis using directional statistics is not a summary statistics of each track but a measurement of diffusional properties of the fiber bundle connecting a pair of ROIs. The statistics of each voxel along the fiber track are summarized by many points using directional statistics along the fiber bundle. Fiber bundle skeletonization and normalization of PDD over tract cross sections allows for error correction and noise cancelation that might arise from tractography artifacts or misalignment. This should also be valid for trajectory changes of tracts under disease-related conditions, as long as a prominent disfiguration or an abnormal morphological change caused by a tumor deviation does not severely divert the alignment of the fiber bundles. In such a case, a lot of false positives may affect the model along the fiber bundles, hindering the correct estimation of PDDs along the actual but diverted tract.

During the second set of performance tests, the results of TPDS and NBS are compared to see whether these methods report the differences between the healthy and MDD populations consistently. We found that most of the right hemisphere-specific connectivity differences reported earlier in MDD have been detected by both of these approaches. The results are much more consistent among the shorter tracts such as frontal connections of the anterior cingulate. However, TPDS reveals additional connectivity differences mainly among longer tracts such as those between temporal and occipital cortex as well as those that contain areas with low FA values and higher crossing fibers such as the amygdala, hippocampus, and thalamus. Another strength of TPDS is due to its revelation about weights, which indicate the amount of difference between the subject populations along the tracts.

These findings are also consistent with MDD models proposed by Drevets et al. (2008) and Mayberg (2003) where MDD can be defined through a limbic–cortical dysregulation model. In this model, the limbic–thalamo–cortical (LTC) circuits, involving the amygdala, thalamus, and orbital and medial PFC, and the limbic–cortical–striatal–pallidal–thalamic (LCSPT) circuits are mainly the affected areas. These connections are found be affected both using NBS and TPDS. Additionally, TPDS revealed temporal, parietal, and occipital cortex connections that are different in MDD. Mainly the differences on inferior fronto-occipital tracts can be also supported by other DTI studies that report significantly decreased FA values among MDD patients (Cheng et al., 2014).

The ROIs reported to have statistically significant connectivity differences in MDD versus healthy participants are consistent with the two well-known lateralization models of emotion. According to the right hemisphere hypothesis, the right hemisphere is dominant in processing emotions (Alves et al., 2008). On the other hand, the valence hypothesis posits that the left hemisphere processes positive (or approach-related) information, but the right hemisphere processes negative (or avoidance-related) information (Alves et al., 2008). Within the context of MDD, hypoactivity in the left hemisphere fronto-striatal loops indicates the lack of downregulation of the subcortical areas. In Figure 9, TPDS—but not NBS—reported differences in the connectivity of the left hemisphere, amygdala, thalamus, and hippocampus, consistent with the valence hypothesis. However, the abundant presentation of right hemisphere ROIs in Figure 9 supports the right hemisphere hypothesis indicating that the connectivity within the right hemisphere may be a biomarker for MDD. TPDS revealed a larger right hemisphere network which was sidestepped by NBS. This network is predominantly composed of the basal temporal lobe structures as well as occipital ROIs such as precuneus and pericalcarine. The difference in the temporal and parietal functionality in MDD is reported less in comparison with those in front striatal structures; however, there is a growing body of literature that focuses on the hypoactivity of the right hemisphere temporal areas in MDD (Bruder et al., 2017). The detection of such ROIs by TPDS is supportive of these studies reported in Bruder et al. (2017). Finally, several rsfMRI biomarkers of MDD are reported in Drysdale et al. (2017). After clustering these biomarkers through machine learning techniques, four different subtypes of MDD can be derived, based on four different clusters of ROIs. Unfortunately, the temporal areas of the brain are excluded in this study, due to a lack of data collection from several participating research sites. However, the ROI network reported by both NBS and TPDS in Figure 9 is also reported in Drysdale et al. (2017), verifying our results in a much larger sample size.

In their meta-analysis of over 231 patients with MDD and 261 comparison participants, Yi Liao et al. found four consistent locations of decreased FA: white matter in the right frontal lobe, right fusiform gyrus, left frontal lobe, and right occipital lobe. Mainly, the right inferior longitudinal fasciculus, right inferior fronto-occipital fasciculus, and right posterior thalamic radiation were involved in such changes (Liao et al., 2013). This covers most of the connection pairs we have found in Figure 9, especially the right fusiform gyrus connections with R. Inferior temporal, parahipppocampal, and temporal gray matter are important because the NBS method failed to reveal all of these areas consistent with the meta-analysis.

In another meta-analysis (Wen et al., 2014), reduced FA is reported in the DLPFC and UF of patients with late-life depression (Wen et al., 2014). Those regions are part of frontostriatal and limbic networks consistent with our findings in Figure 9. This is also consistent with NBS analysis, especially the connections colored in green.

Another recent meta-analysis study has analyzed WM anisotropy and diffusivity in 1,305 MDD patients and 1,602 healthy controls (age range 12–88 years) from 20 samples worldwide (van Velzen et al., 2020). On adults, lower FA was observed in 16 of the 25 ROIs. The largest changes have been found mainly in the anterior corona radiata (ACR), corona radiata (CR), corpus callosum (CC), genu of the corpus callosum (GCC), body of the corpus callosum (BCC), and anterior limb of the internal capsule (ALIC). Significantly lower FA was also observed in the superior fronto-occipital fasciculus (SFO), sagittal stratum (SS), internal capsule (IC), posterior corona radiata (PCR), superior corona radiata (SCR), inferior fronto-occipital fasciculus (IFO), fornix/stria terminalis (FXST), external capsule (EC), and cingulate gyrus of the cingulum bundle (CGC). It is quite important to note that most of these regions are better fitted by TPDS in comparison with TBSS and VBA as revealed by our first test on these methods. The superior fronto-occipital fasciculus (left–right), sagittal stratum (left–right), superior corona radiata (left–right), posterior corona radiata (left–right), superior fronto-occipital fasciculus (left–right), inferior fronto-occipital fasciculus (left–right), external capsule (left–right), fornix (cres)/stria terminalis (left–right), and cingulum (left–right) are all better modeled using TPDS. This is also true for the anterior and superior corona radiata where only the right anterior corona radiata is modeled better with TBSS skeleton. The parts of the corpus callosum are on the other hand fitted better as the genu of the corpus callosum for TPDS, the body of the corpus callosum for VBA, and the splenium of the corpus callosum for TBSS. Overall, the benefit of TPDS is demonstrated in two different ways: 1. By fitting the underlying structural connections to an analytical model in a better way 2. By capturing wider network connectivity differences especially along longer tracts.

## Conclusion

To conclude, we have shown that by analyzing PDDs using directional statistics, more insight is gained about fiber tracts regarding differences between populations. While other connectivity-based analysis methods may disregard the differences between longer fibers, TPDS becomes more robust as fiber tract length increases. In areas with low FA values, the distribution of PDDs among the fiber tracts can differentiate connectivity-based dysfunctions better, due to the power of directional statistics. The directional statistics analysis suggested here can also be applied by augmenting the existing methods, namely TBSS and VBA. Such an addition to the existing methods is valuable because it opens up the possibility to use parametric fitting along with directional statistics. The proposed method could be extended considering second and third directions of the diffusion tensor. In a future study, this can be modeled separately, fitting different distribution models for each direction and analyzing the statistical changes of each direction in disease conditions.

When we implemented TPDS in two subject populations, one healthy and the other with MDD, we found several WM tract differences that are not reported in other methods such as NBS and TBSS. It is imperative to use TPDS on other subject populations and with more subjects to justify its strength in comparison with other methods that perform WM tract-based group analysis.

## Data Availability Statement

The data analyzed in this study is subject to the following licenses/restrictions: The data was collected from medication naive patients for a larger project. The entire project data will be released when the main research is published. Requests to access these datasets should be directed to DG, didemgokcay@gmail.com.

## Ethics Statement

The studies involving human participants were reviewed and approved by Ankara university faculty of medicine clinical research ethics committee. The patients/participants provided their written informed consent to participate in this study.

## Author Contributions

MM designed the model, computational framework, and carried out the implementation. DG directed the study on the MDD population and designed the data collection pipeline. Both authors analysed the data, discussed the results, and contributed to the final manuscript.

## Conflict of Interest

The authors declare that the research was conducted in the absence of any commercial or financial relationships that could be construed as a potential conflict of interest.

## Footnotes

1 TUBITAK 1001, no: 109E081, Ethical board approval: Ankara University Medical College.

2 A black point is called a *border point* if it is six-adjacent to at least one white point. A black point is called an *end point* if it has exactly one black 26-neighbor. Black point *p* is simple in (*Z*^3^, 26, 6,*B*) if and only if all the following conditions hold (Palágyi et al., 2001): (1) The set *N*_26_(*p*)∩(*B*\{*p*}) is not empty (*p* is not an isolated point). (2) The set *N*_26_(*p*)∩(*B*\{*p*}). is 26-ected (3) The set (Z^3^\*B*)∩*N*_6_(*p*) is not empty (*p* is a border point). (4) The set (*Z*^3^\*B*)∩*N*_6_(*p*) is six-connected in the set (*Z*^3^\*B*)∩*N*_18_(*p*).
